# Long-term functional outcome after surgical repair of cranial cruciate ligament disease in dogs

**DOI:** 10.1186/s12917-014-0266-8

**Published:** 2014-11-19

**Authors:** Sari H Mölsä, Heli K Hyytiäinen, Anna K Hielm-Björkman, Outi M Laitinen-Vapaavuori

**Affiliations:** Department of Equine and Small Animal Medicine, Faculty of Veterinary Medicine, University of Helsinki, Helsinki, Finland

**Keywords:** Cranial cruciate ligament, Surgical outcome, Force plate analysis, Goniometry, Long-term, Dog

## Abstract

**Background:**

Cranial cruciate ligament (CCL) rupture is a very common cause of pelvic limb lameness in dogs. Few studies, using objective and validated outcome evaluation methods, have been published to evaluate long-term (>1 year) outcome after CCL repair. A group of 47 dogs with CCL rupture treated with intracapsular, extracapsular, and osteotomy techniques, and 21 healthy control dogs were enrolled in this study. To evaluate long-term surgical outcome, at a minimum of 1.5 years after unilateral CCL surgery, force plate, orthopedic, radiographic, and physiotherapeutic examinations, including evaluation of active range of motion (AROM), symmetry of thrust from the ground, symmetry of muscle mass, and static weight bearing (SWB) of pelvic limbs, and goniometry of the stifle and tarsal joints, were done.

**Results:**

At a mean of 2.8 ± 0.9 years after surgery, no significant differences were found in average ground reaction forces or SWB between the surgically treated and control dog limbs, when dogs with no other orthopedic findings were included (n = 21). However, in surgically treated limbs, approximately 30% of the dogs had decreased static or dynamic weight bearing when symmetry of weight bearing was evaluated, 40-50% of dogs showed limitations of AROM in sitting position, and two-thirds of dogs had weakness in thrust from the ground. The stifle joint extension angles were lower (*P* <0.001) and flexion angles higher (*P* <0.001) in surgically treated than in contralateral joints, when dogs with no contralateral stifle problems were included (n = 33). In dogs treated using the intracapsular technique, the distribution percentage per limb of peak vertical force (DPVF) in surgically treated limbs was significantly lower than in dogs treated with osteotomy techniques (*P* =0.044).

**Conclusions:**

The average long-term dynamic and static weight bearing of the surgically treated limbs returned to the level of healthy limbs. However, extension and flexion angles of the surgically treated stifles remained inferior to healthy joints, and impairment of AROM and weakness in thrust from the ground in the surgically treated limbs were frequently present. Ground reaction forces may be inadequate as a sole method for assessing functional outcome after cranial cruciate ligament repair.

## Background

Cranial cruciate ligament (CCL) rupture is a common cause of canine pelvic limb lameness and stifle joint osteoarthritis. Surgical treatment is advocated to stabilize the stifle joint, alleviate pain, treat any concurrent meniscal pathology, and decelerate the development of osteoarthritis [1]. Numerous surgical techniques have been developed and can be broadly classified as intracapsular ligament replacements, extracapsular suture techniques and neutralizing dynamic osteotomy techniques [2]. The search for the most beneficial surgical technique is ongoing.

The surgical outcome after CCL surgery has often been evaluated through clinical examination, radiography, and owner assessment. Force plate analysis has been the gold standard for objective evaluation of dynamic weight bearing, and it has also been used to evaluate outcome of CCL repair [3-12]. All studies report significant improvement of dynamic weight bearing after surgery, but the results are more variable when return to the level of healthy limbs is considered. In clinical patients, some studies have reported return of dynamic weight bearing to the level of healthy limbs after extracapsular repair [5] and tibial plateau leveling osteotomy (TPLO) [6,10], whereas in others the ground reaction forces in surgically treated limbs or pelvic limb symmetry indices have remained inferior to full function after repair with extracapsular [6,7,10], intracapsular [7], TPLO [7], and tibial tuberosity advancement (TTA) [11] techniques. Few studies directly comparing different surgical techniques by using force plate analysis exist. Conzemius et al. [7] reported that the results of limb function in Labrador Retrievers treated with extracapsular and TPLO techniques were similar but superior to the intracapsular technique 6 months after surgery. Also in another study, no significant differences were found in limb function between TPLO and extracapsular repair 2 years after surgery [3]. On the other hand, two recent studies indicate that TPLO leads to limb function that is superior to that produced by extracapsular surgery [8,10].

In most dogs, despite the surgical technique chosen, osteoarthritic changes progress after CCL surgery [3,13-15] and this may cause deterioration of the dog’s clinical condition later in life. However, only a few true long-term (>1 year) CCL repair outcome studies, using objective and validated outcome evaluation methods, have been published [3,16,17]. Au. et al. [3] evaluated ground reaction forces, stifle joint range of motion and thigh circumference of the surgically treated limbs 2 years after surgery. Peak vertical forces increased significantly after surgery, but the focus of the paper was to compare surgical techniques, not to evaluate the return of limb function to the preinjury level. However, after initial improvement, stifle joint range of motion started to decrease. Also in another study, stifle joint range of motion and thigh circumference in the surgically treated limbs remained significantly decreased relative to contralateral limbs 1-5 years after surgery [16]. In the third study 30% of the surgically treated dogs were found to have signs of chronic pain when long-term outcome was evaluated at a mean of 2.7 years after surgery using a validated pain index [17].

In veterinary orthopedic patients, the use of rehabilitation by physiotherapists that are specialized in animal physiotherapy has increased substantially during the last decade. Animal physiotherapists often use goniometric measurements, subjective or objective evaluation of muscle atrophy, and evaluation of weight bearing, posture, and thrust from the ground to evaluate the patient’s functional condition and response to given treatment. In cruciate disease research, stifle joint goniometric measurements and thigh circumference measurements have been used to evaluate surgical outcome in several studies [3,8,16,18-23]. However, according to our knowledge, a thorough evaluation of the stifle joints done by a professional animal physiotherapist to assess surgical outcome after CCL surgery has not been reported.

The primary purpose of our study was to evaluate surgical outcome at a minimum of 1.5 years after CCL surgery. This was done by combining force plate analysis, clinical orthopedic and physiotherapeutic evaluations, and radiography. The secondary purpose was to compare surgical techniques used to repair CCL rupture. Our hypothesis was that in the long term, the function of the surgically treated limb would remain inferior to the healthy limb.

## Methods

### Study group

Based on a questionnaire study [17] and an another unpublished questionnaire data, owners were invited to bring their dogs to a clinical evaluation, including general, orthopedic, physiotherapeutic, and radiographic examination and force plate analysis. The inclusion criteria for dogs were surgically treated unilateral CCL rupture, a minimum time interval of 1.5 years between surgery and evaluation, and body mass over 17 kg. Patients with bilaterally treated CCL rupture, other known concomitant stifle problems (i.e. patellar luxation, septic or immune-mediated arthritis), or owner-reported significant orthopedic or neurologic problems, were excluded. In addition, possible nonsteroidal anti-inflammatory drug (NSAID), opioid or corticosteroid pain medication, and nutraceutical and fatty acid supplements were withdrawn at a minimum of 7 days, long-term corticosteroids 30 days, and pentosan polysulphate 90 days before the evaluation. The study protocol was approved by the Ethics Committee of Viikki campus, University of Helsinki, and written consent was obtained from all owners. During the evaluation the members of the research team were unaware of which pelvic limb had been operated on and the surgical technique used.

### Building subgroups

Two additional subgroups were formed. Subgroup 1 dogs were used when force plate, static weight bearing, active range of motion and muscle atrophy results were evaluated. For these evaluations, only study group dogs with no signs of other orthopedic disease were included to minimize the effect of other conditions on the test results. On the other hand, when goniometric results in surgically treated limbs were compared to contralateral limbs, the study group dogs that did not have any findings in the contralateral stifle/tarsal joints, i.e. subgroup 2, were included. Subgroup 2 dogs were allowed to have hip dysplasia, elbow OA etc. because these findings were regarded to have no effect on the results of stifle or tarsal goniometry.

### Control group

For the force plate and physiotherapeutic evaluation, a group of 21 clinically healthy Labrador Retrievers and Rottweilers, voluntarily presented by their owners, was used [24]. Inclusion criteria were: age 1-8 years, no known orthopedic problems, and radiographic screening results free of elbow and hip dysplasia according to the Fédération Cynologique Internationale screening protocol.

### Review of medical records

With permission from the owners, the surgical records were reviewed to ascertain the surgical date, technique used, and the surgically treated limb and to obtain information about the meniscal tear and possible complications or revision surgery needed.

### Orthopedic examination

A full orthopedic examination was done to all dogs and included lameness evaluation on a scale from 0 to 4 (no/mild/moderate/severe weight bearing/non-weight bearing) [25] and palpation of the thoracic and pelvic limbs and spine as well as evaluation of conscious proprioception and withdrawal reflex. The stifle joints were palpated for pain (no/mild/moderate/severe), crepitation (no/mild/moderate/severe), periarticular swelling (no/mild/moderate/severe), decrease in range of motion (ROM) (no/mild/moderate/severe), and patellar luxation (grade 0/1/2/3/4) and also evaluated for a positive tibial compression test (negative/positive) and, under sedation with medetomidine 10-20 μg/kg (Dorbene, Syva, Leon, Spain) and butorphanol 0.1 mg/kg (Butordol, Intervet International, Unterschleissheim, Germany) for the cranial drawer test (no/mild/moderate/severe drawer sign). The spine was evaluated for pain and all other joints for pain, crepitation, swelling, decreased ROM and instability (no/yes). While sedated, an Ortolani test was used to evaluate hip joint laxity (negative/positive).

### Radiography

The stifle, hip, and elbow joints bilaterally as well as the lumbar spine of the study group dogs were radiographed under sedation. The amount of osteoarthritis in stifle joints was evaluated from mediolateral and craniocaudal views (no/mild/moderate/severe) [26] in hip joints from an extended ventrodorsal view (no/mild/moderate/severe), and in elbow joints from 45° flexed mediolateral and craniocaudal views on a scale from 0 to III [27]. In addition, the lumbar spine was evaluated from laterolateral and ventrodorsal views for the amount of spondylosis deformans on a scale from 0 to IV [28], and the amount of osteoarthritis (no/mild or moderate/severe).

### Force plate analysis

Gait was evaluated using a piezoelectric force plate (Kistler force plate, type 9286, Kistler Instrumente AG, Winterthur, Switzerland) embedded in a 14 m runway and computer-based software program (Acquire 7.3, Sharon Software Inc., DeWitt, MI). Three pairs of photoelectric cells, positioned 1 m apart from each other, were used to measure the velocity and acceleration of the dogs. Dogs were guided over the force plate by the owner or a member of the research team at a trotting velocity of 2.10-2.50 m/s and a maximum acceleration of ±0.5 m/s^2^.

Data from five valid trials for each side were normalized for the dog’s body mass and averaged. Peak vertical forces (PVFs) and vertical impulses (VIs) in thoracic and pelvic limbs were evaluated. In addition, distribution percentages per limb of peak vertical forces (DPVFs) was calculated using the equation

$$ \mathrm{D}\mathrm{P}\mathrm{V}{\mathrm{F}}_{\mathrm{STPL}}=100\ \mathrm{P}\mathrm{V}{\mathrm{F}}_{\mathrm{STPL}}/\ \left(\mathrm{P}\mathrm{V}{\mathrm{F}}_{\mathrm{STPL}} + P\mathrm{V}{\mathrm{F}}_{\mathrm{CP}L} + \mathrm{P}\mathrm{V}{\mathrm{F}}_{\mathrm{ITL}} + \mathrm{P}\mathrm{V}{\mathrm{F}}_{\mathrm{CTL}}\right) $$

where PVF_STPL_ is the PVFs recorded in the surgically treated limbs, PVF_CPL_ in the contralateral pelvic limbs, PVF_ITL_ in the ipsilateral thoracic limbs and PVF_CTL_ in the contralateral thoracic limbs. The DPVFs were calculated similarly for contralateral pelvic and thoracic limbs of surgically treated dogs as well as thoracic and pelvic limbs of control dogs.

A symmetry index (SI) [29] was calculated for the pelvic limb PVF and VI in surgically treated and control dogs using the equation

$$ \mathrm{S}\mathrm{I} = 200\ \left({\mathrm{X}}_{\mathrm{CPL}/\mathrm{L}}\hbox{--}\ {\mathrm{X}}_{\mathrm{STPL}/\mathrm{R}}\right)\ /\ \left({X}_{\mathrm{CPL}/\mathrm{L}} + {\mathrm{X}}_{\mathrm{STPL}/\mathrm{R}}\right) $$

where X_CPL/L_ is the value recorded in the contralateral (study group) or left (control group) pelvic limb and X_STPL/R_ is the value recorded in the surgically treated (study group) or right (control group) pelvic limb. In surgically treated dogs, a symmetry index value of 0 indicates perfect symmetry; a positive value indicates decreased dynamic weight bearing on the surgically treated limb and a negative value decreased dynamic weight bearing on the contralateral pelvic limb. The symmetry indices were calculated similarly for control group dogs, but absolute values of the symmetry indices were used. Thus, only the magnitude of the symmetry index value was evaluated, while side (right or left pelvic limb) of deviation from perfect symmetry was disregarded. The control group values were used to determine the cut-off value for differentiation between normal and lame dogs by using the equation: Mean + Standard deviation. Based on the cut-off value, the surgically treated dogs were assigned to have normal gait symmetry if SI was between the negative and positive cut-off values, lame on the surgically treated limb if SI was greater than cut-off value, or lame on the contralateral pelvic limb if SI was lower than negative cut-off value.

### Physiotherapeutic examination

A physiotherapeutic examination was done by a physiotherapist specialized in veterinary physiotherapy, as previously described [30]. The full assessment consisted of a visual evaluation of lameness and possible diagonal movement during it, movement on stairs, functional active range of motion (AROM) and thrust from the ground, manual evaluation of muscle atrophy of pelvic limbs, manual evaluation and quantitative measurement of static weight bearing (SWB), and a measurement of passive range of motion (PROM) in stifle and tarsal joints [30]. Of all the physiotherapeutic examination tasks used, the results of AROM, symmetry of thrust between pelvic limbs, muscle atrophy, quantitative SWB of pelvic limbs, and PROM in stifle and tarsal joints are presented here.

When evaluating functional AROM and symmetry of thrust, the dog was led over a 20-m distance and asked to sit and sit-to-move 3 times, and similarly lie-down and lie-to-move 3 times within equal distances. The position of pelvic limbs during sitting and lying was observed, and possible functional limitations or compensations of the pelvic limbs in sitting or lying position (external rotation or abduction of the limb, and limited flexion of stifle and/or tarsal joints) or weakness of pelvic limbs in thrust from sitting or lying positions were recorded [30]. Subjective manual evaluation of muscle atrophy was done by palpating the symmetry of the muscle bulk of pelvic limbs and recorded as either symmetrical muscle mass in pelvic limbs or decreased muscle mass in either the left or right pelvic limb [30].

Quantitative measurement of SWB in pelvic limbs was done using digital bathroom scales, as previously described [31]. Two identical commercially available bathroom scales were used under each limb and the thoracic limbs were placed on a custom-made platform of the same height as the scales. The owner held the dog from the front, keeping it in a straight, square-standing position and the examiner placed the pelvic limbs symmetrically onto the scales, and recorded the measurements for both limbs. The results of four measurements for each limb were averaged and converted from kilograms to percentages proportional to the dog’s body mass (SWB%). In addition, difference in static weight bearing between the pelvic limbs (DiffSWB%) was calculated using the following equation:

$$ 100\ \left(\mathrm{S}\mathrm{W}{\mathrm{B}}_{\mathrm{CPL}}\left(\mathrm{kg}\right)\ \hbox{--}\ \mathrm{S}\mathrm{W}{\mathrm{B}}_{\mathrm{STPL}}\left(\mathrm{kg}\right)\right)/\ \mathrm{body}\ \mathrm{mass}\ \left(\mathrm{kg}\right) $$

where SWB_CPL_ is the value recorded in the contralateral pelvic limb and SWB_STPL_ the value recorded in the surgically treated limb [31]. Based on the published DiffSWB% cut-off value of 6% in healthy control dogs [31], the surgically treated dogs were divided into groups with symmetrical SWB, decreased SWB on the surgically treated limb, or decreased SWB on the contralateral pelvic limb.

PROM was measured in un-sedated dogs from both stifle and tarsal joints in extension and flexion using a universal goniometer with a five-degree scale. The measurements were taken in lateral recumbency three times and averaged [30]. In addition, the extension and flexion results of the surgically treated limbs were reported as percentages of the contralateral limbs. Because flexion angles had a tendency to be smaller (higher in number) in the surgically treated limbs, for calculation of percentages the flexion angles were subtracted from 180°.

### Statistical analysis

Statistical analysis was done using the SPSS software program (version 21 for Windows; SPSS Inc., Chicago, IL). Kolmogorov-Smirnov and Shapiro-Wilk tests were applied to determine whether the data were normally distributed. Results from nominal and ordinal data (surgical technique, condition of the meniscus, stifle joint palpation and radiographic findings, AROM, muscle atrophy), symmetry index and DiffSWB% are reported as frequencies and percentages. For continuous data (age at time of surgery, body mass at time of evaluation, follow-up, force plate data, SWB%, PROM), the results are summarized as mean ± SD, and for age, body mass, and follow-up also as range.

The Kruskall-Wallis test with pairwise comparisons was used to evaluate differences in age, follow-up, stifle joint palpation findings, and amount of radiographic osteoarthritis between surgical techniques, and Independent samples t-test or one-way ANOVA was used to evaluate differences in body mass between study group and control dogs or surgical techniques. The Mann-Whitney rank sum test for non-normally distributed and Independent samples t-test for normally distributed force plate, SWB%, and PROM data were used to evaluate differences between surgically treated limbs and contralateral limbs (force plate, SWB%, PROM) or control dog limbs (force plate, SWB%). Similarly, the Kruskall-Wallis test with pairwise comparisons for non-normally distributed and one-way ANOVA with Bonferroni and Tukey post hoc analyses for normally distributed force plate, SWB%, and PROM data were used to evaluate differences between surgical techniques. *P* <0.05 was considered significant.

## Results

### Study group

From the 278 questionnaire results, 47 dogs (25 females, 22 males) with owners willing to bring their dog to the evaluation fulfilled the inclusion criteria. The breeds of these dogs were Labrador Retriever (15), Rottweiler (6), mixed breed (4), Golden Retriever (3), Bernese Mountain Dog (2), Doberman Pincher (2), Newfoundland Dog (2), Nova Scotia Duck Tolling Retriever (2), and one each of Beauceron, Black Russian Terrier, Bordeaux Dog, Bullmastiff, Collie, Dalmatian, Giant Schnauzer, Karelian Bear Dog, Short-Haired German Pointer, South African Boerboel and Wheaten Terrier. The mean ± SD (range) age of the dogs and their body mass at the time of evaluation were 6.9 ± 2.7 (1.8-13.0) years and 38.2 ± 9.5 (17.5-60) kg, respectively.

Of the pelvic limbs (31 left, 16 right), 19 limbs (40.4%) had been surgically treated using a modification of the original intracapsular technique [32], with an autograft that was passed over the top of the lateral femoral condyle, 7 limbs (14.9%) using variations of the original modification of the extracapsular technique [33], with a nylon leader line or polyester sutures, and 21 limbs (44.7%) using the TPLO (9) [34], the TTA (7) [35], or triple tibial osteotomy (TTO, 5) [18] techniques. Diagnosis of the CCL rupture and inspection of the joint had been done via arthrotomy in all dogs. The condition of the meniscus was reported in 23 dogs (48.9%), with damage in 10 and an intact meniscus in 13 dogs. Postoperative complications were reported in two dogs. One dog had loosening of the screws after TPLO surgery and was treated successfully with revision surgery, and the other dog had excessive scar tissue formation in the surgical wound after intracapsular repair. Mean ± SD (range) time interval between surgery and evaluation visit (follow-up) was 2.8 ± 0.9 (1.5-4.4) years. No significant difference was present between the surgical technique groups in the age of the dogs or body mass of the dogs at the time of evaluation. The mean follow-up was significantly shorter in the osteotomy technique group (2.2 ± 0.6 years) than in the intracapsular (3.3 ± 0.8 years, *P* =0.001) or extracapsular (3.6 ± 0.4 years, *P* =0.002) technique groups.

### Control group

The mean ± SD age and body mass of the healthy control dogs (14 females, 7 males) were 3.2 ± 1.6 years and 35.5 ± 8.2 kg, respectively. The healthy control dogs were significantly younger than the dogs in the study group (*P* <0.001). No significant difference was found in body mass between the healthy control group and study group dogs.

### Orthopedic and radiographic examination

At follow-up, grade 1/4 lameness of the surgically treated limb was seen in 8 dogs (3 intracapsular, 1 extracapsular, 4 osteotomy). One dog treated with the intracapsular technique had grade 2/4 lameness. In addition, 7 dogs had signs of lameness in the contralateral pelvic limb (grade 1/4 in 6 dogs, grade 2/4 in one dog) and 2 dogs in both front limbs (grade 1/4). The gait could not be evaluated in one surgically treated dog due to lack of cooperation. The 21 healthy control dogs did not show any signs of orthopedic disease or lameness.

Orthopedic and radiographic findings in the surgically treated stifle joints are presented in Table 1. The radiographs were unavailable for one dog. The severity of stifle joint crepitation was significantly lower in the osteotomy group than in the extracapsular (*P* =0.030) or intracapsular (*P* =0.028) group, and amount of periarticular swelling was significantly lower in the osteotomy than in the extracapsular group (*P* =0.047). No significant differences were found between surgical techniques in pain response to stifle flexion/extension. Of 12 dogs with positive drawer sign, 5 had been treated with intracapsular and 7 with osteotomy techniques. Tibial compression test was negative in all stifle joints. The amount of radiographic osteoarthritis was lower in the osteotomy group than in the intracapsular group (*P* =0.007).

**Table 1:** **Stifle joint findings of the surgically treated limb; orthopedic and radiographic examination**

	**Severity of stifle joint findings**			
	**No findings**	**Mild**	**Moderate**	**Severe**
**Pain response to stifle flexion/extension (n = 47)**	68.1% (32)	25.5% (12)	6.4% (3)	-
**Periarticular swelling (n = 47)**	-	36.2% (17)	53.2% (25)	10.6% (5)
**Crepitation (n = 47)**	6.4% (3)	34.0% (16)	51.1% (24)	8.5% (4)
**Cranial drawer sign (n = 39)**	69.2% (27)	15.4% (6)	12.8% (5)	2.6% (1)
**Radiographic OA (n = 46)**	2.2% (1)	19.6% (9)	45.6% (21)	32.6% (15)

Results are expressed as percentages (number) of dogs. n, number of dogs evaluated; OA, osteoarthritis.

In the contralateral stifle joints clinical findings were recorded in 9 dogs, including crepitation in all dogs, periarticular swelling in 8, pain in 3, and decreased ROM in 2 dogs. Radiographic signs of osteoarthritis were seen in 11 dogs, and drawer sign was positive in 3 dogs. Concurrent clinical findings were also seen uni- or bilaterally in hip joints of 17 dogs, elbow joints of 8 dogs, and shoulder joints of 6 dogs. Pain on palpation of the spine was found in 19 dogs. Radiographic findings of osteoarthritis were seen uni- or bilaterally in the hip joints of 21 dogs, elbow joints of 4 dogs, and the lumbosacral spine of 13 dogs.

Based on orthopedic and radiographic examinations, 21 dogs were included in subgroup 1 and 33 dogs in subgroup 2.

### Force plate analysis

In 6 dogs the force plate analysis could not be performed due to lack of cooperation or inability to maintain adequate trotting speed. Mean ± SD trotting velocity and acceleration of the study group (n = 41), subgroup 1 (n = 21) and control group dogs (n = 21) were 2.25 ± 0.07, 2.27 ± 0.07 and 2.26 ± 0.06 m/s, and - 0.05 ± 0.12, - 0.09 ± 0.13 and - 0.05 ± 0.14 m/s^2^, respectively. Body mass, trotting velocity or acceleration did not differ significantly between the study group or subgroup 1 and the control group dogs, between the surgically treated and the contralateral limbs or between the surgical technique groups.

When force plate results for the study group were evaluated, no significant differences emerged when comparing the surgically treated limb with the contralateral pelvic limb or with the pelvic limbs of control dogs.

In subgroup 1, surgically treated limbs had significantly lower PVF (*P* =0.040) and DPVF (*P* =0.005) than contralateral pelvic limbs, but no significant differences were found when surgically treated limbs were compared with control dog limbs (Table 2). The symmetry index cut-off values, 7.1% for PVF and 8.1% for VI were rounded up to 8% and 9%, respectively, to improve the specificity of the cut-off value. Based on the cut-off values, the symmetry index for PVF was <8% in 13 dogs (61.9%), indicating normal gait symmetry, and ≥8% in 7 dogs (33.3%), indicating lameness. In one dog, the symmetry index indicated lameness of the contralateral pelvic limb (≤ -8%). Similarly, the symmetry index for VI was <9% in 15 dogs (71.4%), indicating normal gait symmetry, and ≥9% in 6 dogs (28.6%), indicating lameness. Of the 7 dogs in which one or both symmetry indices indicated lameness, 3 had been treated with intracapsular, 2 with extracapsular and 2 with osteotomy techniques.

**Table 2:** **Dynamic and static weight bearing in subgroup 1, surgical technique groups and control dogs**

	**Subgroup 1 (n = 21)**				**Intra (n = 7)**		**Extra (n = 3)**		**Osteo (n = 11)**		**Control group (n = 21)**	
	**CTL**	**ITL**	**STPL**	**CPL**	**STPL**	**CPL**	**STPL**	**CPL**	**STPL**	**CPL**	**TL**	**PL**
**Force plate analysis**												
**PVF (%N/N)**	117.8 (7.2)	117.1 (6.7)	70.6 (7.0)#	75.3 (7.3)	67.2 (7.6)	75.1 (6.7)	NA	NA	73.2 (6.8)	75.2 (8.9)	121.4 (10.2)	72.2 (4.0)
**VI (%Ns/N)**	16.2 (1.5)	16.1 (1.5)	8.7 (1.2)	9.4 (1.0)	8.3 (0.9)#	9.5 (1.0)	NA	NA	9.2 (1.3)	9.5 (1.1)	16.2 (1.1)	8.7 (0.8)
**DPVF (%)**	31.0 (1.1)	30.8 (1.1)	18.5 (1.3)#	19.8 (1.4)	17.8 (1.5)#*	19.9 (1.3)	NA	NA	19.2 (0.9)	19.7 (1.7)	31.3 (1.1)	18.7 (1.1)
**Static weight bearing**												
**SWB%**	NE	NE	15.5 (5.6)#	19.7 (3.6)	13.8 (7.3)#	21.2 (3.3)	NA	NA	16.8 (5.0)	19.4 (3.9)	NE	17.7 (2.8)

Results are expressed as mean (SD). #Significant (P <0.05) difference present between the surgically treated and contralateral pelvic limbs. *Significant (P <0.05) difference present in the measured value between the limbs treated with intracapsular and osteotomy techniques.

CTL, contralateral thoracic limb; ITL, ipsilateral thoracic limb; STPL, surgically treated pelvic limb; CPL, contralateral pelvic limb; TL, thoracic limb; PL, pelvic limb, PVF, peak vertical force; VI, vertical impulse; DPVF, percentage distribution of peak vertical forces on each limb; SWB%, static weight bearing reported as percentages proportional to body mass; NA, not analyzed due to the low number of dogs in extracapsular group; NE, SWB% is not evaluated for thoracic limbs.

When force plate results of different surgical technique groups were evaluated, no significant differences were found in study group between intracapsular, extracapsular and osteotomy techniques, or either in study group or subgroup 1 when surgically treated limbs in each technique group separately were compared with pelvic limbs in control dogs. Subgroup 1 dogs that had been treated with the intracapsular technique had significantly lower DPVF in the surgically treated limb than the dogs treated with osteotomy techniques (*P* =0.044). The dogs that had been treated with the intracapsular technique (n = 7) had significantly lower VI (*P* =0.035) and DPVF (*P* =0.018) in the surgically treated limb than in their contralateral pelvic limb. No significant differences were seen when limbs treated with the osteotomy technique (n = 11) were compared with their contralateral pelvic limbs. The number of dogs treated with the extracapsular technique (n = 3) was too low to be analyzed.

### Physiotherapeutic examination

In visual evaluations of functional AROM, the most frequent findings were in the sitting position. In subgroup 1, decreased flexion of the tarsal joint was seen in 12 dogs (57.1%) (2 intracapsular, 10 osteotomy), decreased flexion of the stifle joint in 10 dogs (47.6%) (2 intracapsular, 1 extracapsular, 7 osteotomy), and abduction of the limb in 9 dogs (42.9%) (1 intracapsular, 1 extracapsular, 7 osteotomy). Except for one dog, the positive findings were always localized in the surgically treated limbs. Thrust from the sitting position was weaker in the surgically treated limb in 14 dogs (66.7%) (3 intracapsular, 1 extracapsular, 10 osteotomy). When evaluating muscle atrophy in subgroup 1, altogether 16 dogs (76.2%) had decreased muscle mass of the surgically treated pelvic limb on palpation (5 intracapsular, 2 extracapsular, 9 osteotomy), while only 4 dogs (19.0%) had symmetrical muscle mass (2 intracapsular, 1 extracapsular, 1 osteotomy), and one dog (4.8%) had decreased muscle mass in the contralateral pelvic limb.

In 2 dogs the SWB could not be performed due to lack of cooperation. When results of SWB were evaluated, all dogs in both the study group and subgroup 1 had a significantly lower SWB% in the surgically treated limb than in the contralateral pelvic limb (*P* =0.010 and *P* =0.006, respectively), but no significant differences were found when surgically treated limbs were compared with control dog limbs (Table 2). Based on the previously reported 6% cut-off value in DiffSWB%, of 21 dogs, 13 (61.9%) had symmetrical weight bearing, 6 (28.6%) had decreased weight bearing in the surgically treated limb, and 2 dogs (9.5%) bore less weight on the contralateral pelvic limb. Of the dogs with decreased weight bearing in the surgically treated limb, 3 had been treated with intracapsular and 3 with osteotomy techniques.

In the study group, no significant differences emerged when SWB in surgically treated limbs was compared between surgical techniques or separately in each surgical technique group with either contralateral or control dog limbs. Similarly, in subgroup 1 dogs, no significant differences were found between surgical techniques. When each surgical technique group in subgroup 1 was evaluated separately and surgically treated limbs were compared with the contralateral and control dog limbs, no significant differences were observed in the osteotomy group (n = 11), but in the intracapsular group (n = 7) the SWB% in the surgically treated limb was significantly lower than that in the contralateral limb (*P* =0.030) (Table 2). The number of dogs in the extracapsular group (n = 3) was too low to be analyzed.

The PROM was not evaluated in 3 dogs due to lack of cooperation and in one dog due to concurrent orthopedic findings in the tarsal joint. When PROMs of surgically treated limbs in subgroup 2 were compared with contralateral limbs, stifle extension angles (*P* <0.001) were significantly lower and stifle flexion (*P* <0.001) and tarsal flexion (*P* =0.002) angles were significantly higher (loss of flexion) in the surgically treated limb (Table 3). Similar differences were present also in the intracapsular and osteotomy groups when each surgical technique was evaluated separately and the surgically treated limb was compared with the contralateral limb. In the extracapsular group, stifle flexion angles (*P* =0.045) were significantly higher (loss of flexion) than in contralateral limbs. No significant differences were found between the surgical techniques.

**Table 3:** **Stifle and tarsal joint flexion and extension in subgroup 2 and in surgical technique groups**

	**Subgroup 2 (n = 33)**			**Intra (n = 11)**			**Extra (n = 6)**			**Osteo (n = 16)**		
	**STPL(°)**	**CPL(°)**	**%**	**STPL(°)**	**CPL(°)**	**%**	**STPL(°)**	**CPL(°)**	**%**	**STPL(°)**	**CPL(°)**	**%**
**Stifle joint**												
**Ext**	149.2 (8.6)*	160.3 (6.6)	93.1	148.2 (7.5)*	163.0 (2.5)	90.9	144.7 (10.9)	155.3 (11.1)	93.2	151.7 (8.0)*	160.3 (5.6)	94.6
**Flex**	47.9 (7.2)*	41.1 (6.5)	95.1	48.0 (5.4)*	40.3 (8.5)	94.5	49.7 (5.2)*	44.2 (2.0)	95.9	47.1 (8.9)*	40.4 (6.0)	95.2
**Tarsal joint**												
**Ext**	173.0 (7.5)	172.9 (6.3)	+0.1	176.5 (5.4)	174.1 (5.8)	+1.4	174.4 (6.3)	173.3 (6.8)	+0.6	170.0 (8.2)	172.0 (6.8)	98.8
**Flex**	46.7 (11.9)*	37.6 (9.1)	93.6	42.4 (10.8)*	32.6 (6.0)	93.5	52.0 (12.7)	43.6 (10.7)	93.8	47.7 (12.1)*	38.9 (8.9)	93.8

Results are expressed as mean (SD).*Significant (P <0.05) difference in the measured angle between the surgically treated limb and contralateral pelvic limb. STPL, surgically treated pelvic limb; CPL, contralateral pelvic limb. In percentage results, positive (+) value indicates higher extension angles in surgically treated limbs than in contralateral limbs.

## Discussion

We evaluated overall long-term surgical outcome at a mean of 2.8 years after CCL repair using a combination of force plate analysis, orthopedic and radiographic examinations and as a new aspect, a physiotherapeutic evaluation performed by a veterinary physiotherapist.

### Force plate analysis

Force plate analysis showed no significant differences in ground reaction forces (PVF and VI) between the surgically treated limbs and the pelvic limbs of control dogs as well as in VI between the surgically treated and the contralateral limbs. However, the PVF of surgically treated limbs was significantly lower than the corresponding value for the contralateral pelvic limbs. The mean (SD) difference in PVF between the surgically treated and contralateral limbs was 4.7% (7.9), but it must be taken into account that load distribution from the injured to the contralateral pelvic or other limbs often occurs and artificially increases the difference between limbs [36]. Furthermore, the PVF of the surgically treated limbs was only 1.6% (8.5) lower than the corresponding value for the control dog limbs and mean VIs were exactly the same (SD 1.5) in surgically treated and control dog limbs.

When the differences are interpreted, the clinical importance of changes must be considered. Our results indicate that the mean differences between the surgically treated and healthy limbs were minor and dynamic weight bearing of the surgically treated limbs had returned to the level of healthy limbs. Similar long-term studies have not been reported before, but these results are in accordance with those of previous short- and mid-term force plate studies where the function of the surgically treated limb was at the level of the contralateral or control dog limbs 7 months after an extracapsular repair [5], or 4 and 12 months after TPLO surgery [6,10].

Although the average differences in ground reaction forces between surgically treated and healthy limbs were minor, the SIs for PVF and VI were greater than cut-off values in almost one-third of dogs, indicating clinically relevant problem of weight bearing in these dogs and revealing considerable variation between the evaluated dogs. By using the SIs, more differences were revealed than what was discovered when the mean ground reaction forces between the surgically treated and healthy limbs were compared.

### Physiotherapeutic examination

A physiotherapeutic examination concentrating on the stifle joints and performed by a veterinary physiotherapist was included in our study. To our knowledge, evaluation of AROM, thrust of pelvic limbs, or SWB has not been used to evaluate surgical outcome after CCL repair. Interestingly, compensations of AROM were seen in 40-50% of the dogs. In addition, the compensations of AROM were almost always localized to the surgically treated limb, not to the contralateral healthy limb.

Static weight bearing of the pelvic limbs was measured using commercial bathroom scales, and validity study of their results has been reported elsewhere [31]. The mean (SD) SWB% of the surgically treated limbs was 4.2% (8.5) lower than that of the contralateral limbs, reaching statistical significance. It can be assumed that, similar to dynamic weight bearing, load distribution to other limbs occurs also when measuring SWB. The difference between surgically treated and control dog limbs was only 2.2% (6.2) and not statistically significant. Similar to the variation seen in dynamic weight bearing, in almost one-third of dogs also the DiffSWB% indicated decreased static weight bearing in the surgically treated limb.

Goniometry of the stifle joints has been evaluated after CCL repair in several studies [3,8,16,18-23]. CCL rupture causes pain and disuse of the affected limb, resulting in decreased stifle ROM and muscle atrophy [23]. The stifle ROM improves after surgical repair [3,8,18,20,22], and the improvement can be enhanced by intense postoperative physiotherapy [23]. However, in some studies the stifle joint ROM has been shown to decrease with time after surgery [3,8,18,22]. Au et al. [3] reported that stifle joint ROM angles in surgically treated limbs were below the preoperative values 2 years after TPLO or extracapsular repair. Our results are also in accordance with another study, in which significant loss of extension and flexion of the surgically treated stifle joint, compared with the contralateral joint, was reported 1-5 years after TPLO surgery [16]. The most probable cause for the decrease of ROM over time is the progression of osteoarthritis, causing functional impairment due to pain and mechanical restriction of joint mobility due to fibrous tissue and new bone formation in the joint and its periarticular tissues. The clinical relevance of a loss of 5% in flexion and 7% in extension of the stifle joint ROM in our study is unknown. Jandi and Schulman [21] reported that loss of flexion or extension ≥10° was associated with higher lameness scores, but we did not find any correlation between goniometric results and dynamic or static weight bearing.

In addition to loss of extension and flexion of the stifle joint, also flexion of the tarsal joint was significantly decreased in the surgically treated limbs. Loss of flexion in the tarsal joint might be explained by the anatomical fact that in order to measure full flexion of the tarsus a full flexion of the stifle joint is required and thus the limitations in the stifle joint flexion also may affect ipsilateral tarsal joint flexion angles.

### Pain

An interesting aspect in the long-term assessment of CCL repair is the occurrence of pain. In our study, in almost one-third of dogs the symmetry indices for PVF and VI indicated lameness and the DiffSWB% indicated decreased weight bearing in the surgically treated limb, likely not only reflecting the functional outcome, but also pain in the surgically treated limb. Moreover, in the orthopedic examination, lameness was seen in almost 20% and pain response on flexion and/or extension of the surgically treated stifle joints in one-third of dogs. These findings resonate well with our previous report of chronic pain after CCL surgery, which is based on a validated questionnaire filled out by 253 dog owners and includes the 42 dogs serving as subjects here. In that report, almost one-third of the dogs had signs of chronic pain at a mean of 2.7 years after CCL surgery [17]. Recognition of chronic pain can be challenging for owners [37], and these dogs may often go undiagnosed and untreated. It’s therefore important for clinicians to be aware that CCL-deficient dogs may experience chronic pain, and owners should be educated about recognizing signs of pain so that pain management can be initiated promptly.

### Limitations

There are limitations in our study. Our patient material was very heterogeneous, with many confounding factors. Due to the retrospective study design we were unable to control such variables as conformation and age of the dog, duration of clinical signs before surgery, completeness of the cruciate ligament tear, meniscal pathology, and treatment or postoperative rehabilitation and medications. However, our patient material can be used to assess general surgical outcome and it better reflects a real-life situation with heterogeneity in patient material, surgical procedures, and postoperative treatments. The confounding factors may nevertheless have caused bias in the comparison of surgical techniques. Therefore the results must be interpreted with some caution.

The number of patients was unfortunately low. Due to the limitations set by the force platform, only dogs with a body mass exceeding 17 kg were included. For force plate and physiotherapeutic evaluation, we only included dogs with no other concurrent orthopedic problems (subgroups 1 and 2) in order to minimize the effect of concomitant problems on dynamic or static weight bearing, AROM, and PROM. Although only the dogs with unilaterally treated CCL rupture and no other owner-reported significant orthopedic or neurologic problems were originally included in the study group, during the evaluation surprisingly many of them were diagnosed with bilateral stifle joint pathology or other concurrent orthopedic problems, resulting in a low number of dogs for final evaluation. To detect a statistical significance in PVF between the surgically treated and control dogs using the data generated from this study, 400 dogs would have been needed (using α =0.05 and 80% power). On the other hand, it may be speculated that even if a statistical significance had been shown, a difference of 1.6% in PVF between the surgically treated and control dog limbs might anyway not be clinically significant.

In force plate analysis, we decided to compare the ground reaction forces to both contralateral and control dog limbs, both of which have some disadvantages. Due to weight redistribution to the contralateral pelvic limb or thoracic limbs, the use of the contralateral pelvic limb as a reference has been questioned [36]. On the other hand, when force plate results are compared with data obtained from other individuals, in addition to controlling the velocity, acceleration, and body mass of the dog, the conformation and size of the dogs have an effect on ground reaction forces [24,38,39]. In our study, there were no significant differences in body mass of the dogs between the groups, but although there were many Rottweilers and Labrador Retrievers in the study group, it also included several other breeds with different body conformations, while the control group of healthy dogs included only Rottweilers and Labradors. As a compromise, we compared the results with both contralateral and control dog limbs, and after taking into account the weight distribution, the results were in accordance with each other. Similar comparisons were made for static weight bearing.

In the physiotherapeutic examination, only a subjective assessment of muscle mass symmetry was used when evaluating muscle atrophy. We could have improved the test by measuring atrophy with a tape measure, thus quantifying the measurement and increasing the objectivity of the method. On the other hand, in a recent study [30], the subjective evaluation of muscle atrophy was shown to be sensitive for detecting functional pelvic limb problems. Interestingly, 16 of the 21 dogs evaluated in our study (76%) had decreased muscle mass in the surgically treated limb.

The postoperative treatment did not include any special rehabilitation program and only one of the dogs had had postoperative rehabilitation by a physiotherapist. Previous studies have shown that postoperative rehabilitation significantly improves the surgical outcome [23], and nowadays, rehabilitation is recommended to all CCL-deficient dogs. However, at the time the dogs in our study were treated, postoperative rehabilitation was not yet widely used.

### Comparison of surgical techniques

The dogs treated with osteotomy techniques had less crepitation in the surgically treated limb than dogs treated with intra- or extracapsular techniques. In addition, the amount of radiographic osteoarthritis was lower in the osteotomy group than in the intracapsular group. These results must be interpreted with caution because of potential initial preoperative differences in the orthopedic or radiographic examination between the surgical technique groups. On the other hand, it could be speculated that the preoperative differences in radiographic osteoarthritis between groups might level off in the long run. Also, in two recent studies the preoperative amount of osteoarthritis was reported to correlate negatively with the progression of osteoarthritis postoperatively, meaning that dogs with severe pre-existing osteoarthritis would have less potential for osteoarthritis to worsen, compared with dogs with only minor pre-existing osteoarthritis [40,41].

The low number of dogs treated with extracapsular technique did not allow comparison of ground reaction forces or SWB of this group to other technique groups. The ground reaction forces and SWB% in the osteotomy group had a tendency to be higher than in the intracapsular technique and to reach control dog values. The dogs that had been treated with the intracapsular technique had significantly lower DPVF in the surgically treated limb than the dogs treated with the osteotomy technique. No other significant differences emerged between surgical technique groups when dynamic and static weight bearing or goniometric results were evaluated. No significant differences between surgically treated and contralateral or control dog limbs were found in the osteotomy group, whereas in the intracapsular group statistically significant differences in VI, DPVF and SWB between the surgically treated and contralateral limbs were achieved. Parallel results have been reported also in two previous force plate studies. Conzemius et al. [7] stated that the limb function after intracapsular repair was inferior to limb function after TPLO surgery 6 months postoperatively. Also in another experimental study the limb function after intracapsular repair remained significantly lower than the preoperative function at a 20-week follow-up [9]. In goniometry, stifle joint extension and flexion as well as tarsal joint flexion angles were decreased relative to the contralateral limb both in intracapsular and osteotomy technique groups.

## Conclusions

The average long-term dynamic and static weight bearing of the surgically treated limbs returned to the level of healthy limbs. However, when symmetry of weight bearing was evaluated using cut-off values, approximately 30% of dogs had decreased static or dynamic weight bearing in the surgically treated limb, and almost one-third of dogs showed pain in stifle joint palpation. In addition, the extension and flexion angles of the surgically treated stifles remained inferior to healthy joints, and many dogs showed impairment of AROM and weakness in thrust from the ground in the surgically treated limbs. In sum, measurement of ground reaction forces may be inadequate as a sole method for assessing functional outcome and thus more objective outcome assessment methods should be included in evaluation of CCL repair.

## Abbreviations

AROM: Active range of motion
CCL: Cranial cruciate ligament
CPL: Contralateral pelvic limb
CTL: Contralateral thoracic limb
DiffSWB%: Difference in static weight bearing between the pelvic limbs
DPVF: Percentage distribution of peak vertical force
ITL: Ipsilateral thoracic limb
L: Left
OA: Osteoarthritis
PL: Pelvic limb
PROM: Passive range of motion
PVF: Peak vertical force
R: Right
ROM: Range of motion
SI: Symmetry index
STPL: Surgically treated pelvic limb
SWB: Static weight bearing
SWB%: Static weight bearing as percentages proportional to dogs body mass
TL: Thoracic limb
TPLO: Tibial plateau leveling osteotomy
TTA: Tibial tuberosity advancement
TTO: Triple tibial osteotomy
VI: Vertical impulse
